# Impact of biological rhythms on the importance hierarchy of constituents in time-dependent functional brain networks

**DOI:** 10.3389/fnetp.2023.1237004

**Published:** 2023-08-29

**Authors:** Timo Bröhl, Randi von Wrede, Klaus Lehnertz

**Affiliations:** ^1^ Department of Epileptology, University of Bonn Medical Center, Bonn, Germany; ^2^ Helmholtz Institute for Radiation and Nuclear Physics, University of Bonn, Bonn, Germany; ^3^ Interdisciplinary Center for Complex Systems, University of Bonn, Bonn, Germany

**Keywords:** functional brain network, vertex centrality, edge centrality, circadian rhythm, electroencephalographic signals

## Abstract

Biological rhythms are natural, endogenous cycles with period lengths ranging from less than 24 h (ultradian rhythms) to more than 24 h (infradian rhythms). The impact of the circadian rhythm (approximately 24 h) and ultradian rhythms on spectral characteristics of electroencephalographic (EEG) signals has been investigated for more than half a century. Yet, only little is known on how biological rhythms influence the properties of EEG-derived evolving functional brain networks. Here, we derive such networks from multiday, multichannel EEG recordings and use different centrality concepts to assess the time-varying importance hierarchy of the networks’ vertices and edges as well as the various aspects of their structural integration in the network. We observe strong circadian and ultradian influences that highlight distinct subnetworks in the evolving functional brain networks. Our findings indicate the existence of a vital and fundamental subnetwork that is rather generally involved in ongoing brain activities during wakefulness and sleep.

## 1 Introduction

While describing natural complex dynamical systems is a notoriously difficult endeavor, the network approach (Boccaletti et al., 2006; 2014; Newman, 2018) has been repeatedly shown to provide novel and important insights into such systems in various research areas ranging from neurosciences (Bullmore and Sporns, 2009; Lehnertz et al., 2014) via genomics (Tyler et al., 2009) and proteomics (Uetz et al., 2000) to ecology (Hegland et al., 2009; Olesen et al., 2011; Delmas et al., 2019; Halekotte and Feudel, 2020), climatology (Donges et al., 2009; Zhou et al., 2015), and sociology (Onnela et al., 2007; Palla et al., 2007). This broad applicability is not least explained by the large manifold of network metrics, describing global aspects to local aspects in network terms, which in principle can be directly related to the properties of the described system. Identifying key network constituents is highly relevant when it comes to improving the understanding and control of networks, as it allows us to gain insights about the importance hierarchy of its constituents with respect to the network structure and dynamics. The characterization of a constituent’s role in the network structure and dynamics can be achieved through different concepts and a growing number of metrics such as centralities. Most of these concepts focus on the description of vertices or groups of such [e.g., hubs (Newman, 2003), hub regions in the brain (Stanley et al., 2013; Chung, 2019), and k-core decompositions (Kong et al., 2019)], while only a few metrics assess the centrality of an edge. We recently proposed a novel strength-based edge-centrality concept (Bröhl and Lehnertz, 2022) and introduced modifications to vertex closeness and vertex eigenvector centrality concepts yielding corresponding edge centrality concepts (Bröhl and Lehnertz, 2019). We demonstrated that these edge centralities—together with edge betweenness centrality (Freeman, 1977; Girvan and Newman, 2002)—provide additional information about the network constituents for various topologies. These four centrality concepts, while different in their definition, can be considered complementary in the description of a constituent’s structural integration in the network.

Many early studies assumed networks to be static; however, the recent paradigm shift toward time-dependent (or evolving) networks (Holme and Saramäki, 2012; Kivelä et al., 2014) allows one to describe many systems more accurately. This particularly holds true for biological networks, such as the brain, for which time dependencies on different scales have been observed. Both exogenous and endogenous biological rhythms are expected to assert influences on the level of the network description (Kuhnert et al., 2010; Lehnertz et al., 2017; Mitsis et al., 2020; Kurth et al., 2021; Lehnertz et al., 2021) and therefore on network metrics such as centralities (Geier et al., 2015; Geier and Lehnertz, 2017; Lehnertz et al., 2017; Lehnertz et al., 2021).

For more than 50 years, it has been known that the circadian rhythm and ultradian rhythms impact electroencephalographic (EEG) signals [see Lehnertz et al. (2021) for a recent overview]. Many former studies, however, were based on EEG recordings that either assessed the dynamics of few brain regions only or/and covered timescales ranging only from few seconds to hours. Here, we extend the recent studies and observations (Spoormaker et al., 2011; Chu et al., 2012; Park et al., 2012; Liu et al., 2015; Farahani et al., 2021) and investigate how biological rhythms, particularly the circadian rhythm (with a period length of approximately 24 h), influence the importance hierarchies of the constituents of evolving functional brain networks. Therefore, we focus on both the networks’ vertices that are associated with the sampled brain regions and networks’ edges that represent time-evolving interactions between brain regions.

## 2 Materials and methods

### 2.1 Data

We analyzed electroencephalographic signals obtained from eight subjects (three females, age 19–81 years) with (five subjects) and without disorders (three subjects) of the central nervous system (CNS). All subjects were under stable CNS medication (if taking any). The EEG data were recorded continuously over 4 to 8 days from 19 electrodes placed according to the 10–20 EEG system (Klem et al., 1999) (Cz served as a physical reference) with a sampling rate of 256 Hz, using a 16-bit analog-to-digital converter (Micromed, S.p.A., Mogliano Veneto, Italy). Data were band-pass filtered offline (bandwidth: 1–45 Hz; fourth-order Butterworth characteristic), and a notch filter (third order) was used to suppress contributions at the line frequency (50 Hz). Data used in this study were visually inspected to remove segments containing strong artifacts (e.g., subject movements or amplifier saturation).

### 2.2 Deriving evolving functional brain networks

Time-dependent, fully connected, weighted functional brain networks were constructed through a time-resolved synchronization analysis of an EEG recording (Mormann et al., 2000; Osterhage et al., 2007; Kuhnert et al., 2010; Goodfellow et al., 2022) to track the changes in the importance hierarchies of network constituents (Geier and Lehnertz, 2017; Rings et al., 2019; Fruengel et al., 2020) possibly related to biological rhythms (Lehnertz et al., 2021). To perform this, network vertices were associated with brain regions whose dynamics were sampled by electrodes and network edges were associated with time-varying estimates of the strength of interactions between the dynamics of the pairs of those brain regions, regardless of their anatomical connections. As an estimate of the strength of the interaction, we employed mean phase coherence (*R*) (Mormann et al., 2000), which assesses the degree of synchronization between two phase time series (*R* = 1 indicates fully phase-synchronized brain regions, and *R* = 0 indicates no phase synchronization). A non-overlapping sliding window with a duration of 20  s (5,120 data points) was used to calculate *R* in a time-resolved manner. The chosen duration of a window can be considered a compromise between the required statistical accuracy for the calculation of *R* and the approximate stationarity within the window’s length (Lehnertz et al., 2017). For each window, we derived the instantaneous phase time series via the Hilbert transform of the EEG time series. An important property of this analytic signal approach (particularly in the case of two or more superimposed oscillatory components) is that the instantaneous frequency relates to the predominant frequency in the Fourier spectrum (Boashash, 1992). The predominant frequency may be subjected to fluctuations in the EEG time series. Thus, the instantaneous frequency can vary rhythmically around the predominant frequency, which results in spurious estimates of the instantaneous phase. By taking the temporal average, these effects can be reduced. From an electrophysiological point of view, we consider it more reasonable to look adaptively (e.g., via the Hilbert transform) at interactions between predominant rhythms in EEG rather than to look at interactions in some *a priori* fixed frequency bands (e.g., via wavelet transform) for which there is no power in the time series (Osterhage et al., 2007; Frei et al., 2010). Following these steps of analysis for each window, we end up with a temporal sequence of snapshot functional brain networks, each of which consists of *V* vertices and *E* edges and can be described by a weight matrix $W\in {\left[0,1\right]}^{V\times V}$ , where $W_{ij}$ refers to the edge weight (strength of the interaction) between vertices *i* and *j*. The number of actual windows per subject depended on the respective recording duration, thus yielding approximately 9,500–26,000 windows.

### 2.3 Estimating the importance of network constituents

In order to further investigate the temporal sequence of snapshot functional brain networks, different approaches may be adopted. Estimating distance or (dis-)similarity between two networks might be one such approach, although finding suitable distance metrics still remains a challenge (Mheich et al., 2020). Another approach consists of the so-far insufficiently studied concept of multilayer networks (De Domenico, 2017). Yet, due to some fundamental limitations, a meaningful interpretation of multilayer brain networks remains to be explored (Buldú and Papo, 2018). Therefore, here, we pursue the investigation of the time series of networks’ characteristics (Lehnertz et al., 2014; 2017) and utilize the following centrality metrics to estimate importance of each network’s vertices and edges.

Strength centrality of a vertex *i* is the sum of the weights of all edges connected to this vertex:

$$C_{v}^{S}\left(i\right)=\overset{V}{\underset{j=0}{\sum }}W_{ij}.$$ (1)

The higher the vertex’s degree/strength, the more central it is considered to be. A related metric for edges is nearest-neighbor centrality, which considers an edge to be more central when its weight is larger and the strengths of the vertices that are connected by that edge are more similar and higher. Nearest-neighbor centrality of an edge *k* between vertices *a* and *b* is defined as follows (Bröhl and Lehnertz, 2022):

$$C_{e}^{S}\left(k\right)=\frac{C_{v}^{S}\left(a\right)+C_{v}^{S}\left(b\right)-2w_{k}}{|C_{v}^{S}\left(a\right)-C_{v}^{S}\left(b\right)|+1}\;w_{k},$$ (2)

where $k\in \left\{1,\ldots ,E\right\}$ , (*a*, *b*) ∈ {1, *…*, *V*}, and $w_{k}=W_{ab}$ denotes the weight of edge *k* connecting vertices *a* and *b*. Similar to strength centrality of a vertex, nearest-neighbor centrality of an edge is only influenced by its adjacent constituents. Hence, vertices (brain regions) and edges (interactions between pairs of brain regions) that have high $C^{S}$ values are largely interconnected with the neighboring vertices and edges. When structurally viewed, these neighboring constituents would be located in either the same areas of the brain or an adjacent area; from a functional perspective, they might be associated with any area of the brain. Hence, constituents with high $C^{S}$ values are highly interconnected within the functional network, although not necessarily allowing a structural interpretation.

Eigenvector centrality considers the influence of a vertex/edge on the network as a whole, where a vertex/edge is considered central if the vertex/edge adjacent to it is also central, and it is defined as

$$C_{v,e}^{E}\left(i\right)=\frac{1}{\lambda_{\max}}\underset{l}{\sum }M_{il}\;C_{v,e}^{E}\left(l\right).$$ (3)

In case of vertices, (*i*, *l*) ∈ {1, *…*, *V*} and **M** denotes the weight matrix $W^{\left(v\right)}\in {\left[0,1\right]}^{V\times V}$ , where $W_{il}^{\left(v\right)}$ denotes the weight of an edge between vertices *i* and *l*. We define $W_{ii}^{\left(v\right)}:=0\;\forall \;i$ with $i\in \left\{1,\ldots ,V\right\}$ . In the case of edges, (*i*, *l*) ∈ {1, *…*, *E*} and **M** denotes the weight matrix $W^{\left(e\right)}\in {\left[0,1\right]}^{E\times E}$ , whose entries $W_{il}^{\left(e\right)}$ are assigned to the average weight of edges *i* and *l*, if these edges are connected to the same vertex, and 0 otherwise. As mentioned previously, we define $W_{ii}^{\left(e\right)}:=0\;\forall \;i$ with $k\in \left\{1,\ldots ,E\right\}$ . Equation 3 is applied iteratively until eigenvector centrality values remain stable. Hence, eigenvector centrality can be considered a strength-based centrality concept, which, due to its recursive definition, relates a vertex/edge to all the other vertices/edges in the network. Similar to strength/nearest-neighbor centrality, constituents with high $C^{E}$ values are gradiently stronger connected to closer constituents than to those that are far off. Again, distance-related descriptions, such as “close” or “far-off,” relate to the functional network and, hence, do not necessarily allow a structural interpretation, meaning constituents with large $C^{E}$ values are highly connected to the functional network in a rather general sense. This high inter-connectedness refers to many and/or possibly strong interactions with constituents either associated with the same brain area or possibly with any other area.

Closeness centrality considers the distance between a vertex/edge and all the other vertices/edges in the network. A vertex/edge with high closeness centrality is considered central as information obtained from this vertex/edge can reach all the other constituents in the network via short paths and so the vertex/edge can exert a more direct influence on the network. Closeness centrality of vertex *k* is defined as (Bavelas, 1950)

$$C_{v}^{C}\left(n\right)=\frac{V-1}{\sum_{m}d_{nm}},$$ (4)

where $\left(n,m\right)\in \left\{1,\ldots ,V\right\}$ and *d*_*nm*_ represents the length of the shortest path between vertices *n* and *m*, which is calculated as the sum of the inverse of all edge weights on the path. Closeness centrality of edge *k* between vertices *a* and *b* can be defined as (Bröhl and Lehnertz, 2019)

$$\begin{array}{cc} C_{e}^{C}\left(k\right) & =\frac{E-1}{\sum_{i}\left(d_{ia}+d_{ib}\right)}=\frac{E-1}{\frac{1}{C_{v}^{C}\left(a\right)}+\frac{1}{C_{v}^{C}\left(b\right)}}\\ & =\left(E-1\right)\frac{C_{v}^{C}\left(a\right)C_{v}^{C}\left(b\right)}{C_{v}^{C}\left(a\right)+C_{v}^{C}\left(b\right)}, \end{array}$$ (5)

where $k\in \left\{1,\ldots ,E\right\}$ and (*a*, *b*, *i*) ∈ {1, *…*, *V*}. Hence, closeness centrality can be considered a path-based centrality concept, which is therefore influenced by the network as a whole. High closeness centrality points toward a constituent, which is associated with any brain area that is functionally “close” to any other constituent associated with any brain area. Hence, certain parts (with high $C^{C}$ ) of certain brain areas interact strongly with many other parts in the same area, while also interacting with many other brain areas.

Betweenness centrality is a measure of how frequently the shortest path traverses a given vertex/edge. A vertex/edge with high betweenness centrality is considered central because it acts as a bridge between other brain regions. Vertex/edge betweenness centrality (of vertex/edge *i*) can be defined as (Freeman, 1977; Brandes, 2001; Girvan and Newman, 2002)

$$C_{v,e}^{B}\left(i\right)=\frac{2}{F}\underset{n\neq m}{\sum }\frac{q_{nm}\left(i\right)}{G_{nm}},$$ (6)

where $i\in \left\{1,\ldots ,V\right\}$ , $i\in \left\{1,\ldots ,E\right\}$ , and $\left\{n,m\right\}\in \left\{1,\ldots ,V\right\}$ ; *q*_*nm*_(*i*) represents the number of shortest paths between vertices *n* and *m* running through vertex/edge *i*, and *G*_*nm*_ represents the total number of shortest paths between vertices *n* and *m*. The length of a path is calculated as the sum of the inverse of all edge weights on that path. The normalization factor is given as *F* = (*V*−1) (*V*−2) in the case of vertices and *F* = *V*(*V*−1) in the case of edges. Hence, betweenness centrality can be considered a path-based centrality concept, which is therefore influenced by the network as a whole. Constituents with high betweenness centrality are likely to be part of bottleneck-like structures spanning between brain areas, both in a structural and functional sense.

In order to facilitate a qualitative comparision of the results obtained with the different centrality concepts, we utilized centrality value-based importance ranking of constituents (Liao et al., 2017). A vertex/edge with the largest centrality value gets assigned to rank 1. The rank increases in increments of 1 for the vertex/edge with the second largest centrality value, third largest centrality value, etc., yielding an increasing rank as the centrality values decrease. This ranking can be further normalized, yielding a relative ranking with the highest relative rank being 1 (most important) and the lowest relative rank being 0 (least important). Hence, we can deduce an importance hierarchy for the vertices and edges of each snapshot functional brain network.

### 2.4 Characterizing the influence of biological rhythms on the importance of network constituents

The aforementioned steps of analysis provide us with a temporal sequence of vertex/edge importance hierarchies of an evolving functional brain network and enable the investigation of how biological rhythms impact this hierarchy. To this end, we proceed as described in Lehnertz et al. (2021) and estimate the power spectral densities [Lomb–Scargle periodogram (Press et al., 1989)] of the respective temporal sequences. Eventually, we quantified the influence of the circadian rhythm on each such sequence as the portion of power for period lengths in the range of 20–28 h relative to the total power in the range of 1–36 h. We refer to this ratio as *P*_24_ in the following sections.

## 3 Results

**FIGURE 1:**
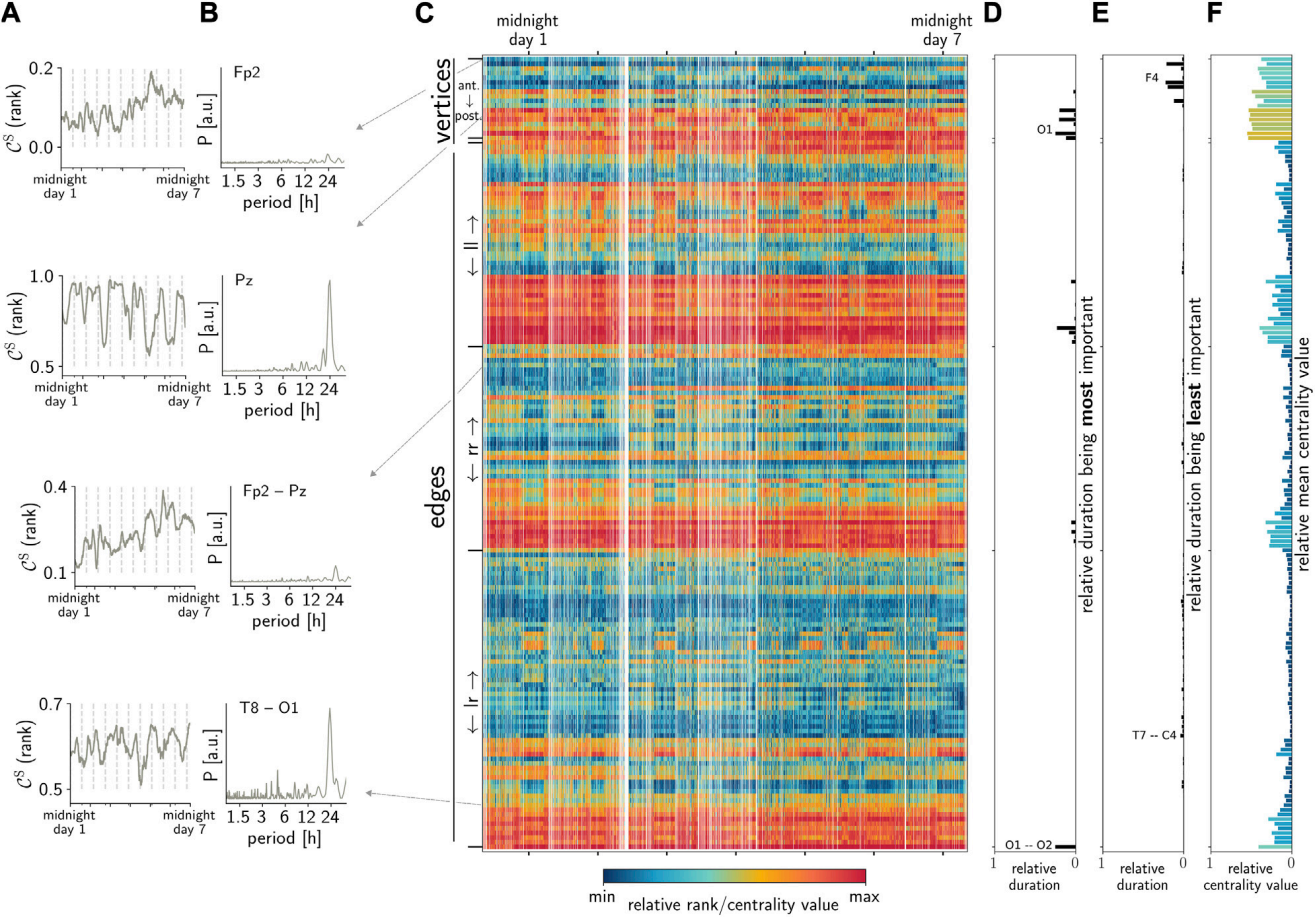
Exemplary observations: **(A)** temporal sequences of the importance of arbitrarily chosen vertices (here, FP2 and PZ) and edges (FP2–PZ and T8–O1) estimated with $C_{v}^{S}$ and $C_{e}^{S}$ , respectively. **(B)** Lomb–Scargle periodograms of sequences in **(A)**. **(C)** Temporal evolution of the relative rank (color-coded) of all network constituents (ant., anterior; post., posterior; ll, left-hemispheric interactions; rr, right-hemispheric interactions; lr, inter-hemispheric interactions; white vertical lines depict recording gaps). Electrode contacts are displayed in the following descending order (top to bottom) respectively: ant. – post.: Fp1, Fp2, F7, F3, Fz, F4, F8, T7, C3, C4, T8, P7, P3, Pz, P4, P8, O1, O2; ll: Fp1-F7, Fp1-F3, Fp1-Fz, Fp1-T7, Fp1-C3, Fp1-P7, Fp1-P3, Fp1-Pz, Fp1-O1, F7-F3, F7-Fz, F7-T7, F7-C3, F7-P7, F7-P3, F7-Pz, F7-O1, F3-Fz, F3-T7, F3-C3, F3-P7, F3-P3, F3-Pz, F3-O1, Fz-T7, Fz-C3, Fz-P7, Fz-P3, Fz-O1, T7-C3, T7-P7, T7-P3, T7-Pz, T7-O1, C3-P7, C3-P3, C3-Pz, C3-O1, P7-P3, P7-Pz, P7-O1, P3-Pz, P3-O1, Pz-O1; rr: Fp2-Fz, Fp2-F4, Fp2-F8, Fp2-C4, Fp2-T8, Fp2-Pz, Fp2-P4, Fp2-P8, Fp2-O2, Fz-F4, Fz-F8, Fz-C4, Fz-T8, Fz-P4, Fz-P8, Fz-O2, F4-F8, F4-C4, F4-T8, F4-Pz, F4-P4, F4-P8, F4-O2, F8-C4, F8-T8, F8-Pz, F8-P4, F8-P8, F8-O2, C4-T8, C4-Pz, C4-P4, C4-P8, C4-O2, T8-Pz, T8-P4, T8-P8, T8-O2, Pz-P4, Pz-P8, Pz-O2, P4-P8, P4-O2, P8-O2; lr: Fp1-Fp2, Fp1-F4, Fp1-F8, Fp1-C4, Fp1-T8, Fp1-P4, Fp1-P8, Fp1-O2, Fp2-F7, Fp2-F3, Fp2-T7, Fp2-C3, Fp2-P7, Fp2-P3, Fp2-O1, F7-F4, F7-F8, F7-C4, F7-T8, F7-P4, F7-P8, F7-O2, F3-F4, F3-F8, F3-C4, F3-T8, F3-P4, F3-P8, F3-O2, Fz-Pz, F4-T7, F4-C3, F4-P7, F4-P3, F4-O1, F8-T7, F8-C3, F8-P7, F8-P3, F8-O1, T7-C4, T7-T8, T7-P4, T7-P8, T7-O2, C3-C4, C3-T8, C3-P4, C3-P8, C3-O2, C4-P7, C4-P3, C4-O1, T8-P7, T8-P3, T8-O1, P7-P4, P7-P8, P7-O2, P3-P4, P3-P8, P3-O2, P4-O1, P8-O1, O1-O2. **(D,E)** Fraction of the recording time during which a respective constituent was the most/least important. **(F)** Average relative centrality values ( $C_{v}^{S}$ and $C_{e}^{S}$ , respectively, normalized to the maximum value) over the recording time.

We observe contributions of rhythms with period lengths of approximately 24 h (and to a lesser extent from rhythms of approximately 12 h or shorter) in all temporal sequences of importance (centrality values and ranks) of the respective network constituents from each subject. However, and contrary to the expectation, we found that these circadian and ultradian contributions are more pronounced in the sequences of some vertices and edges, i.e., some brain regions, as well as the interactions between their dynamics (see Figure 1).

**FIGURE 2:**
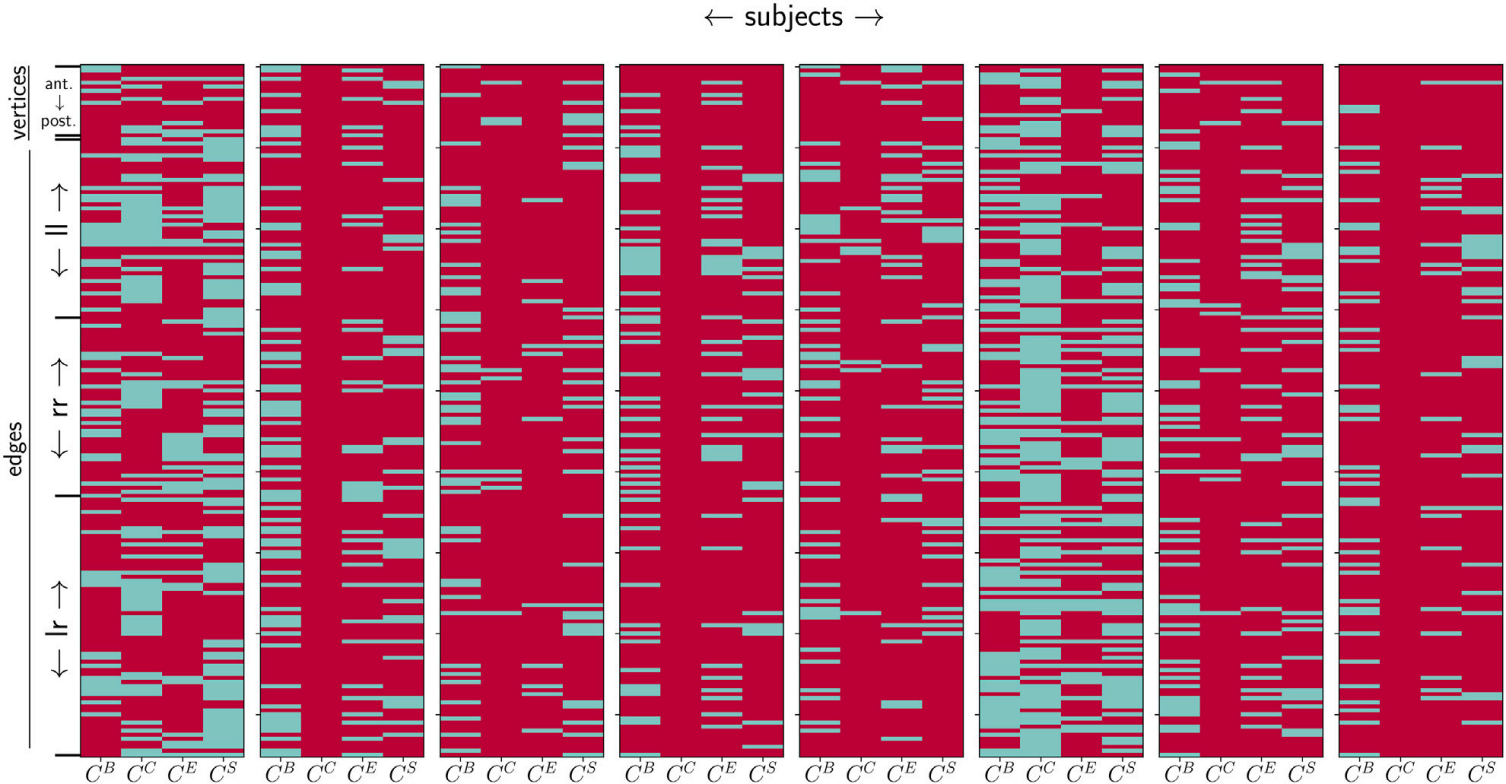
Influence of the circadian rhythm (estimated with *P*_24_) on centrality values of network constituents (assessed with betweenness centrality $C^{B}$ , closeness centrality $C^{C}$ , eigenvector centrality $C^{E}$ , and strength/nearest-neighbor centrality $C^{S}$ ). Pale blue: *P*_24_ ≤ 0.5; red: *P*_24_ > 0.5.

Interestingly, we also observe that the strength of circadian contributions differs for temporal sequences derived with different centrality metrics. This is to be expected, at least to some extent, since the metrics highlight different structural aspects of a network, such as the path-structure or strength distribution. Nevertheless, these observations suggest that the circadian rhythm affects these different structural aspects. Figure 2 demonstrates that this rather unspecific relation regarding path/strength-based centrality metrics for vertices and edges can be observed in the data from all the investigated subjects. Moreover, it becomes quite apparent from this figure that by combining the results yielded by different centrality concepts, almost all network constituents are impacted by the circadian rhythm in all subjects.

**FIGURE 3:**
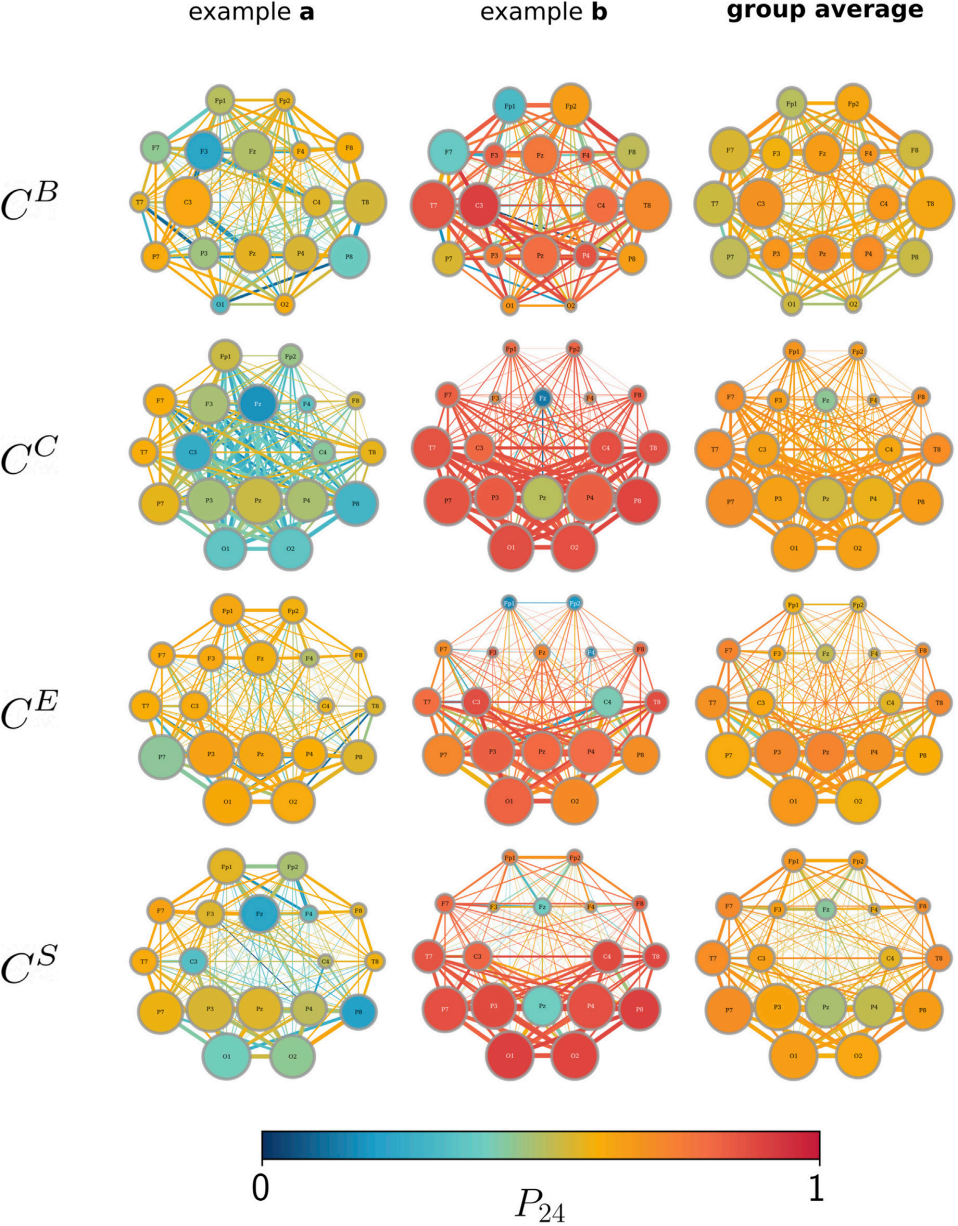
Influence of the circadian rhythm (*P*_24_; color-coded) and the average importance over the recording time (the size of vertices/edges; the larger they are, the more important they are). Importance estimated with betweenness centrality $C^{B}$ , closeness centrality $C^{C}$ , eigenvector centrality $C^{E}$ , and strength/nearest-neighbor centrality $C^{S}$ . Networks are depicted in the layout of the 10–20 EEG system (Klem et al., 1999). Examples a and b (left and middle columns) represent the observed opposing extreme cases from two subjects, either showing an overall little (example a) or strong (example b) influence of the circadian rhythm. The right column shows the group average over all the subjects.

Yet, it can also be observed that there is no trivial relation between the influence of the circadian rhythm (estimated with *P*_24_) and a constituent’s importance (*cf.* Figure 3). Neither for the most nor for the least important network constituents do we observe a generally specific influence of the circadian rhythm (as well as for ultradian rhythms (data not shown)).

**FIGURE 4:**
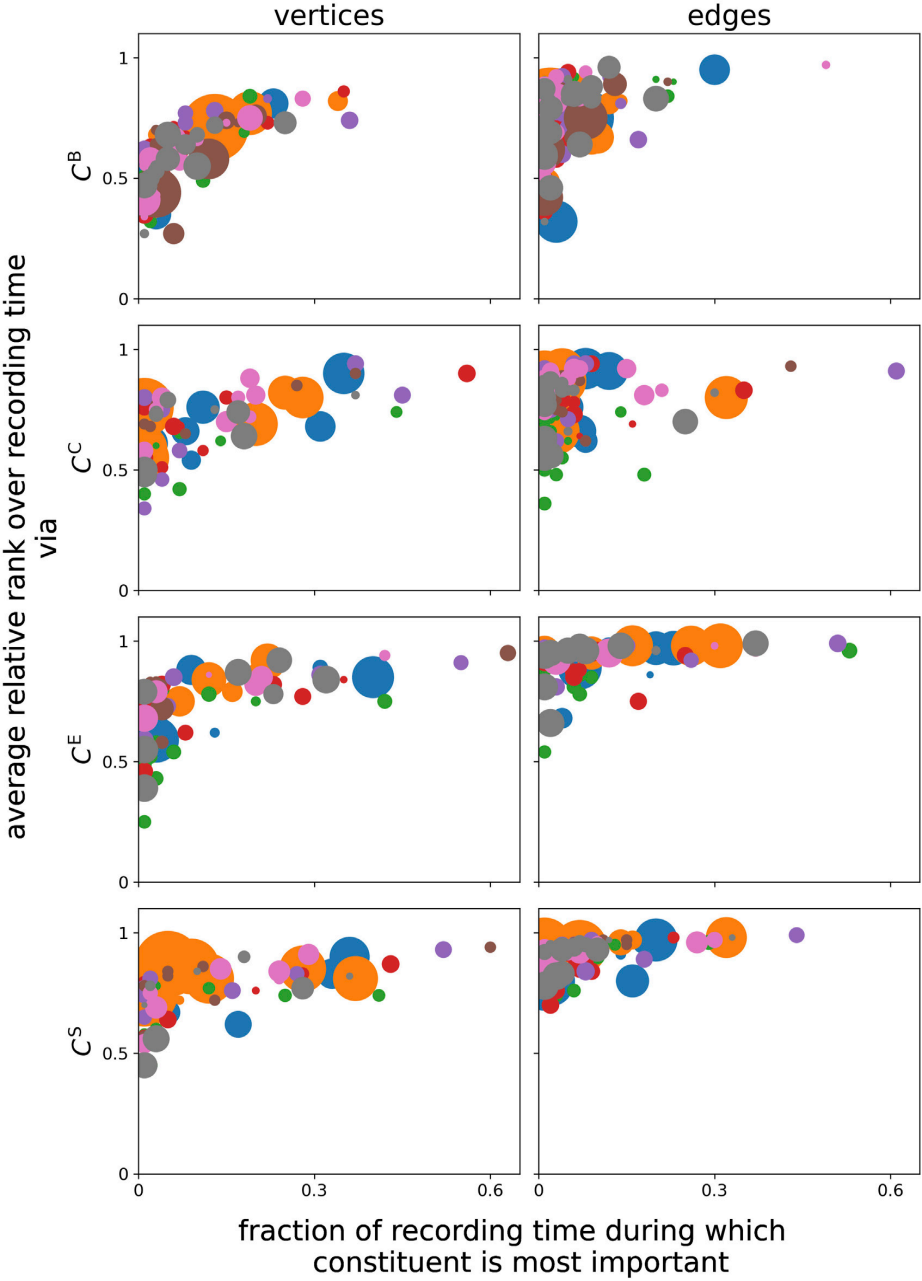
Relation between constituents’ average relative rank over the total recording time and their fraction of the recording time, for which the constituents are deemed the most important. Marker colors encode the different subjects, and marker sizes encode the relative power corresponding to the 24-h peak related to the circadian rhythm.

Furthermore, we find that different centrality metrics identify different constituents as the most important (on average over the whole observation time, in line with previous studies) (see, e.g., Kuhnert et al. (2012); Bröhl and Lehnertz (2019); Bröhl and Lehnertz (2022); and the references therein). One needs to take into account that the constituents deemed the most important on average do not always coincide with the constituents that are deemed the most important for the largest fraction of the recording time (*cf.* Figure 4). Likewise, constituents, for which the temporal profiles of importance are impacted strongly by the circadian rhythm, neither coincide with those constituents that are deemed the most important on average nor with those constituents that are deemed the most important for most of the recording time. This discrepancy cannot be traced back to the ceiling or floor effects, resulting from the definitions of their respective centrality metrics. Overall, we observe a rather unspecific influence of primarily the circadian rhythm on many structural aspects of network constituents: each brain region (vertex) and even interactions between such regions (edges) appear to be influenced, at least to some extent, in their structural integration.

**FIGURE 5:**
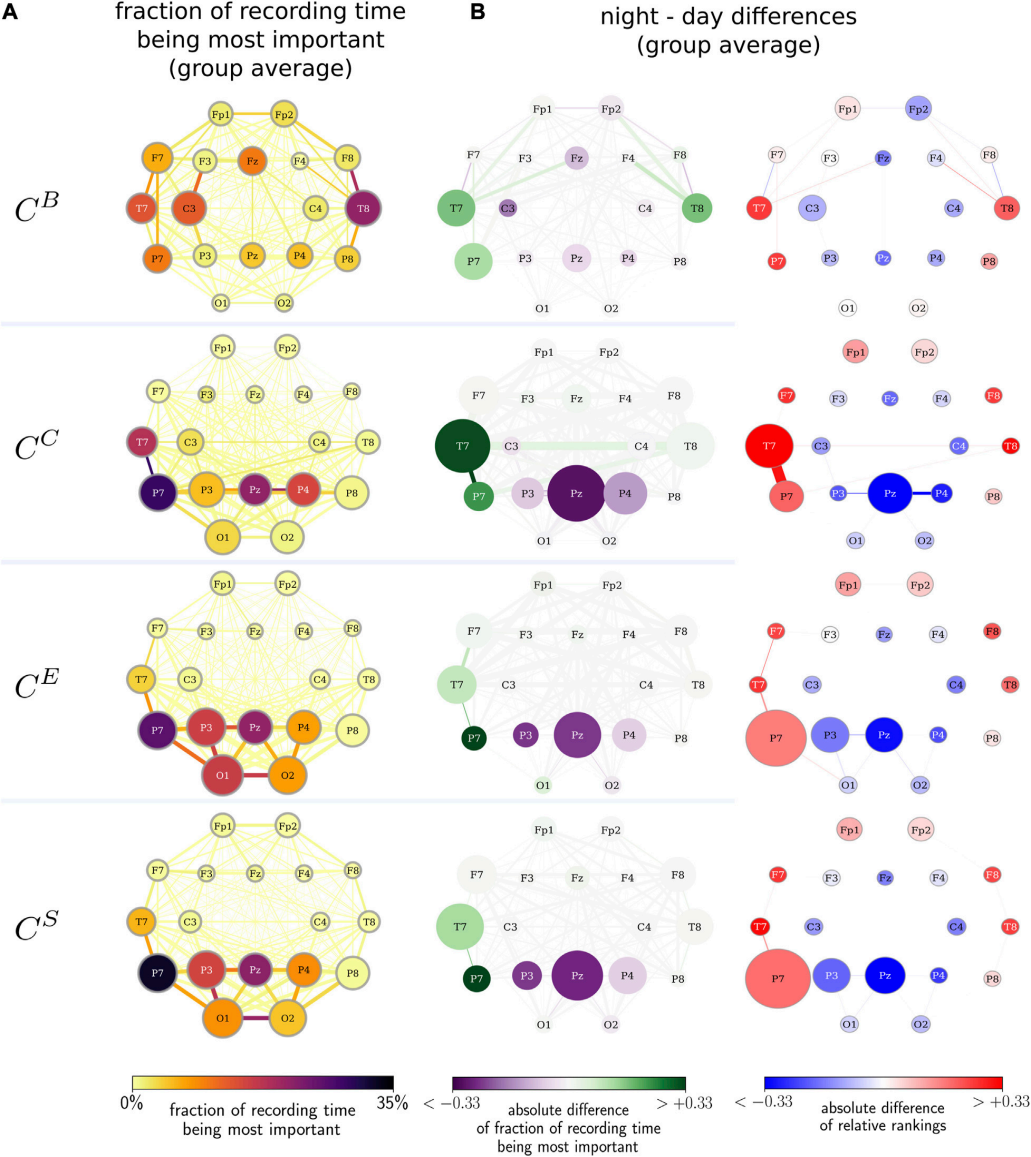
**(A)** Fraction of the recording time during which a network constituent is the most important (color-coded) and the average relative rank of the constituent (size-coded). Importance assessed with betweenness centrality $C^{B}$ , closeness centrality $C^{C}$ , eigenvector centrality $C^{E}$ , and strength/nearest-neighbor centrality $C^{S}$ . Middle column: Absolute night–day differences in the fraction of the recording time during which the respective constituent is the most important (color-coded, green/purple indicating a higher fraction of the recording time during night/day) and the absolute value of the absolute difference in the constituents’ relative rankings (size-coded). **(B)** Absolute night–day difference in constituents’ relative rankings (color-coded, red/blue indicating a higher relative ranking during night/day) and the absolute value of the absolute difference in the fraction of the recording time during which a network constituent is the most important (size-coded). The respective data are averaged over all subjects and their respective night/day periods.

In order to improve on the findings achieved so far, we investigate whether there exists a day/night pattern in the temporal evolution of the importance of vertices and edges (*cf.* Figures 1A, C). Interestingly, we observe that the largest differences in the importance of network constituents between night- and daytimes are related to very distinct brain areas along with their interactions (see Figure 5). These vertices and edges not only exhibit the largest change in centrality values when functional brain networks transit from night- to daytimes but are also further identified as the most important constituent on average and for the largest fraction of the total recording time. While betweenness centrality highlights bilateral frontotemporal vertices and edges, closeness centrality highlights the predominantly left temporoparietal vertices and edges. Both strength-based centralities (eigenvector centrality and nearest-neighbor centrality) predominantly highlight the left temporoparietal and left parietooccipital vertices and edges. Independent of the employed centrality metric, temporoparietal network constituents (vertices T7, T8, and P7 as well as their associated edges) are identified as the most important during daytime (here, 12:00 to 16:00 h). In contrast, during nighttime (here, 24:00 to 4:00 h), the importance shifts to parietal network constituents (vertices P3, P4, and PZ as well as their associated edges). Apart from this night–daytime-related spatial shift of the importance of a few network components, our findings also point toward a key arrangement of connected vertices and edges, relating to a subnetwork that comprises vertices T7, P7, P3 PZ, and P4 together with their associated edges. This subnetwork, which is slightly more dominantly located on the left brain hemisphere, is possibly involved in ongoing activities during wakefulness and sleep.

## 4 Discussion

We investigated how the circadian rhythm impacts the time-dependent importance hierarchy of the vertices and edges of an evolving functional brain network. We employed different path- and strength-based centrality metrics for vertices and edges to comprehensively characterize the importance hierarchy of these network constituents. At the single-constituent level, we observed that their time-dependent changes in importance are clearly modulated by the circadian rhythm although to a varying degree. Irrespective of their interindividual variabilities, we observed pronounced differences in the constituents’ importance hierarchy when contrasting data obtained during daytimes and nighttimes. This may point to a local, circadian rhythm-driven modulation of the dynamics of various brain regions alongside their interactions. These brain regions form vital and fundamental subnetworks within the evolving functional brain networks.

The subnetwork highlighted with betweenness centrality comprises temporofrontal brain regions from both hemispheres. It is rather unexpected that the subnetworks, as highlighted with closeness, eigenvector, and strength/nearest-neighbor centrality metrics (but not with betweenness centrality), are largely overlapping, despite the fact that the different centrality metrics assess different structural aspects of network constituents. This subnetwork is predominantly restricted to the temporoparietal brain regions, with a left-hemispheric dominance during the nighttime. However, whether the subnetworks observed here are related to the resting-state network needs further investigation (Raichle, 2015).

Studies revealed that the hippocampus, deep inside the temporal lobe, and the visual cortex are simultaneously involved in the reactivation of coherent memory traces during sleep, which points toward a contribution to the memory consolidation process (Prabhakaran et al., 2000; Albouy et al., 2013). The interaction between those brain regions might possibly relate to the T7–P7 (–T8) structure in the observed vital subnetworks as these vertices and edges, associated with these electrodes and interactions between the sampled brain regions, are deemed more important in general and for a larger fraction of time during the nighttime compared to the daytime.

During the daytime, the vertices and edges associated with the parietal lobes (PZ, P3, and P4) are deemed more important and for a larger fraction of time compared to the nighttime. These areas consolidate spatial and visual information and integrate perceptions with other sensory inputs, resulting in the recognition of the trajectories of moving objects. These areas also mediate proprioception (perception of the position of the body in space) and are involved in skills such as arithmetic, writing, left–right orientation, and finger perception (see Rizzolatti et al. (1997) for an overview). Since these functions may also be involved during dream phases (which account for approximately 25% of the sleep period), these vertices and edges are nonetheless important during nighttimes although for shorter periods of time.

Overall, we observe that circadian (and ultradian) biological rhythms strongly influence the importance hierarchy, as assessed with different centrality concepts, of the constituents in time-dependent functional brain networks. These observations highlight, for each employed centrality concept, distinct subnetworks in evolving functional brain networks. The structural composition of these networks, however, largely coincides, which points toward the existence of a vital and fundamental subnetwork that is rather generally involved in ongoing brain activities.

## Data Availability

The data analyzed in this study are subject to the following licenses/restrictions: the datasets presented in this article are not readily available because they contain information that could compromise the privacy of research participants. Requests to access the datasets should be directed to the corresponding author.
